# Supplementary material for: The interactome of intact mitochondria by cross-linking mass spectrometry provides evidence for coexisting respiratory supercomplexes
Source: Mol Cell Proteomics. 2017 Dec 8;17(2):216–32. doi: 10.1074/mcp.RA117.000470 (PMC5795388; doi:10.1074/mcp.RA117.000470)
Supplement: Supplemental Data [file supp_RA117.000470_133922_0_supp_23978_fzffwf.zip › spectra_annotation/mito_DR_spectra_annotation/293-1-27-1-155-1.pdf]

NKFGAPQK
IIDAVKAGLEQGSDAGYLAESQK

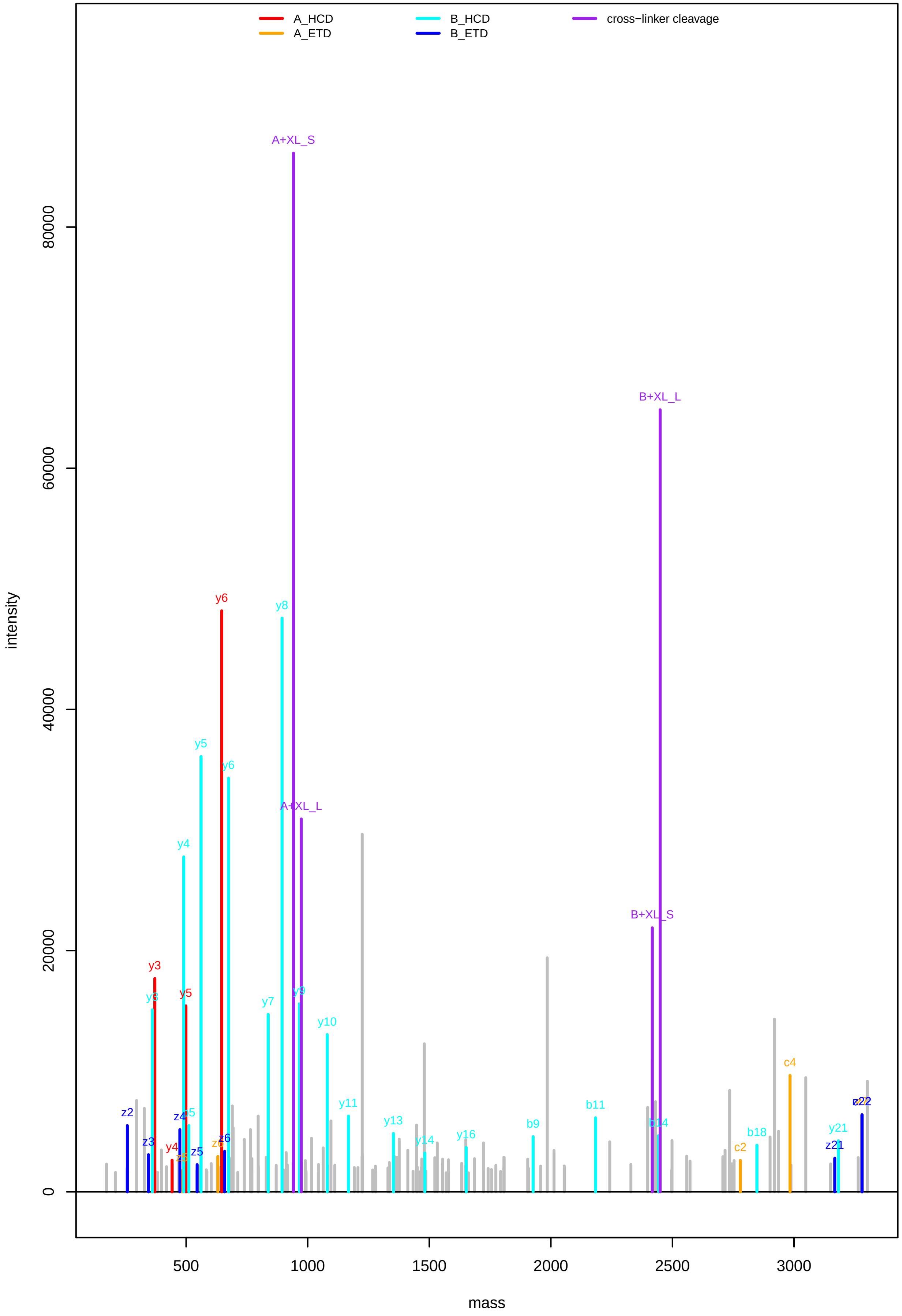

NKFGAPQK+XL_S

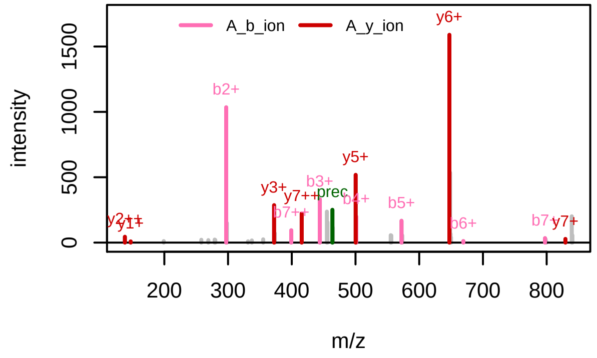

NKFGAPQK+XL_L

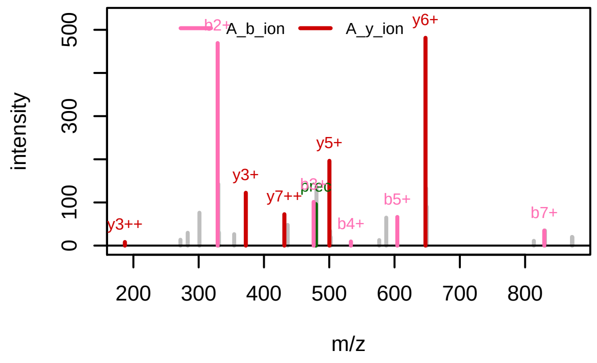

IIDAVKAGLEQGSDAGYLAESQK+XL_S

IIDAVKAGLEQGSDAGYLAESQK+XL_L
